# A rare case of multicentric synchronous bi-frontal glioma in a young female. Diagnostic and therapeutic problems: a case report

**DOI:** 10.1186/1757-1626-2-81

**Published:** 2009-01-23

**Authors:** Maria Cristina Turola, Roberta Schivalocchi, Vania Ramponi, Alessando De Vito, Maria Giulia Nanni, Giovanni Francesco Frivoli

**Affiliations:** 1 Servizio Psichiatrico Diagnosi e Cura AUSL Ferrara, Corso Giovecca 203, 44100 Ferrara, Italy; 2 U.O. Neurochirurgia, Azienda Ospedaliero-Universitaria S. Anna, Corso Giovecca 203 44100 Ferrara, Italy; 3 Unità Operativa Neurologia, Azienda Ospedaliero-Universitaria Ferrara, Corso Giovecca 203, 4410 Ferrara, Italy; 4 Clinica Psichiatrica Università di Ferrara, Corso Giovecca 203, 44100 Ferrara, Italy

## Abstract

Multicentric glioblastoma is a uncommon brain malignant tumour.

We report the case of a 43-years-old woman, born in Ukraine and living in Italy, who manifested an initial isolated epileptic seizure and subsequent atypical psychiatric symptoms. Clinical neurological examination, Brain Computed Tomography and standard EEG examinations were negative at the moment of admission. A month later, she presented apathy, apraxia, psychomotor slowdown and expressive aphasia. A Magnetic Resonance Imaging examination showed a bi-frontal lesion. The patient underwent to two neurosurgical removals of the lesions: histological examination demonstrated the presence of a grade IV glioblastoma.

Clinical onset, diagnostic and therapeutic problems are discussed.

In case of atypical psychiatric presentation, it should be taken into consideration neoplastic, inflammatory or infective causes. Despite the absence of focal neurological signs and basal CT scan and EEG alterations, complementary imaging examinations, such as MRI and contrast enhancement CT, are necessary, especially when the conditions become quickly worse

## Introduction

Multicentric gliomas are well-separated lesions, localized in different lobes or hemispheres, which cannot be ascribed to dissemination through commissural pathways, cerebrospinal-fluid (CSF), blood or local extension. Incidence range is included from 2.3 to 9.1%. Despite of advances in neuroradiological techniques, in case of multicentric cerebral lesions, differential diagnosis may require cerebral biopsy [1-3]. We report the case of 43-year-old female with a bi-frontal grade IV glioma and discuss the clinical onset, diagnostic and therapeutic problems.

## Case presentation

A 43-years-old woman, born in Ukraine, living and working in Italy since 1999 (nine years), came to observation of the First Aid Unit of Ferrara Hospital in March 2008, due to an epileptic seizure with generalized convulsions which occurred while she was working as assistant for a patient of the Pneumology Unit, in the same Hospital. She was found by the nurses of the ward, prone on the floor, unresponsive and presenting diffuse muscular jerks. This episode had spontaneous resolution in a few minutes and the patient completely regained consciousness. No signs of sphincters relaxation or secondary trauma were evident. Her vital parameters never got altered during and immediately after the seizure and she had normal blood pressure and body temperature. The nurses reported appearance, a few hours before the seizure, of a brief, sudden episode of diminished awareness of environment and inability to respond to external stimuli; this was erroneously blamed to tiredness and lack of sleep.

Neither medical therapy at home nor voluntary drug abuse or toxin exposure were reported. She never had epileptic events or other neurological symptoms before. In her past clinical history no significant diseases could be found, except for a surgical intervention of saphenectomy in February 2008. She did not report familiar or personal history of psychiatric or neurological disorders, or, in particular, of epileptic disease.

She was working as an assistant for a disabled lady and had always done an efficient and competent job.

The patient got married when she was twenty-five years old and divorced ten years ago. She had one pregnancy; her daughter is twenty years old. She doesn't smoke and she drinks alcohol only occasionally. She is 165 cm high and she weights 63 kg.

Friends described her as a clever, polite, mild woman, able to speak a good Italian in a short time.

Taken to First Aid Unit and after medical evaluation, the patient was addressed to Brain CT in order to exclude haematomas or vascular injuries; she was then taken to Neurological Unit.

### Neurological Unit

The patient was admitted in the Neurological Unit of Ferrara Hospital due to an acute, apparently generalized, convulsive seizure

Past medical history was not significant. A mild change in her emotional balance and behaviour control was reported in the past few months, when she felt more anxious than usual, with occasional compensative alcohol abuse.

**Figure 1:**
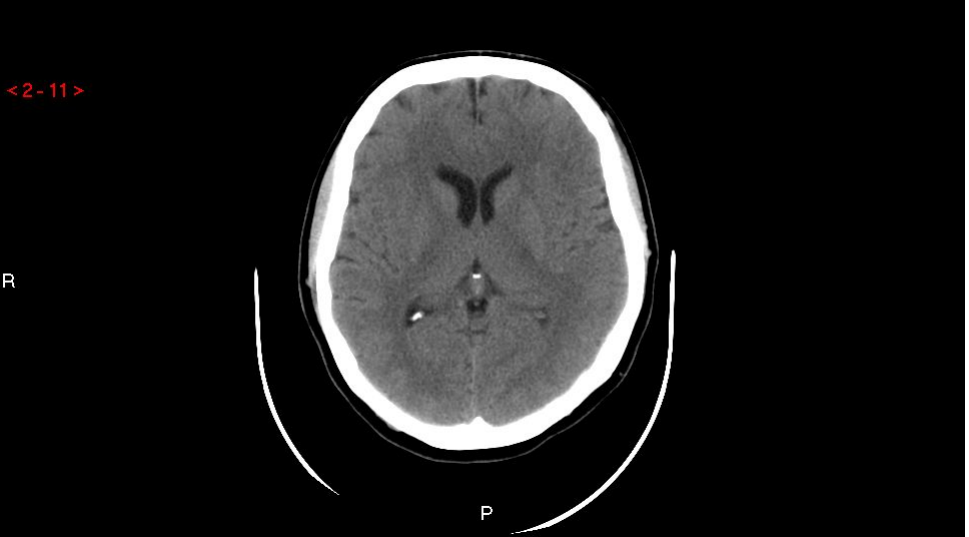
**Brain Computed tomography performed during the first recovery**.

Physical and neurological examinations and Brain Computed Tomography Scan (fig. Fig. 1) on admission were unremarkable as well as the standard Electroencephalogram, which showed a bilateral, symmetrical 10 Hz Alfa rhythm with normal arrest reaction to eyes opening, and did not reveal any epileptic rhythms or figures.

The routine Electrocardiogram and laboratory tests (blood cells count and morphometry, plasmatic glucose level, seric electrolytes, coagulation, kidney and liver function indexes) didn't show any significant abnormalities, except for a mild red blood cells macrocitosis (MCV 97 fl). Blood pressure and temperature were normal.

The patient had no evident epileptic relapse during the subsequent clinical observation.

As the first line routine investigations did not reveal the cause of her epileptic episode, we planned to perform second level diagnostic tests, particularly sleep-deprivation sensitized electroencephalogram and cerebral MRI (Magnetic Resonance Imaging), in order to unravel the epileptic focus or possible organic brain pathology.

Nevertheless, we could not complete our diagnostic program: the patient progressively developed an intense anxious state, explained away with the fear to lose her job because she was ill. For this reason she left the hospital two days after the admission, against physician's opinion.

A month later, the woman visited again the First Aid Unit, taken by her friends, upset by her sudden change in behaviour: she did not speak, even if she was able to understand and respond with gestures and signs; she was slow in all daily activities; she seemed uninterested and emotionless, lost in her world. Since her first visit to Hospital she didn't experienced any epileptic symptom.

After medical examinations, psychiatric and neurological consultations, the patient was hospitalized in a Psychiatric Unit for acute inpatient.

### Psychiatric Unit

The psychiatric diagnostic possibilities were: a psychotic acute crisis, a major depression episode or hysteria.

**Figure 2:**
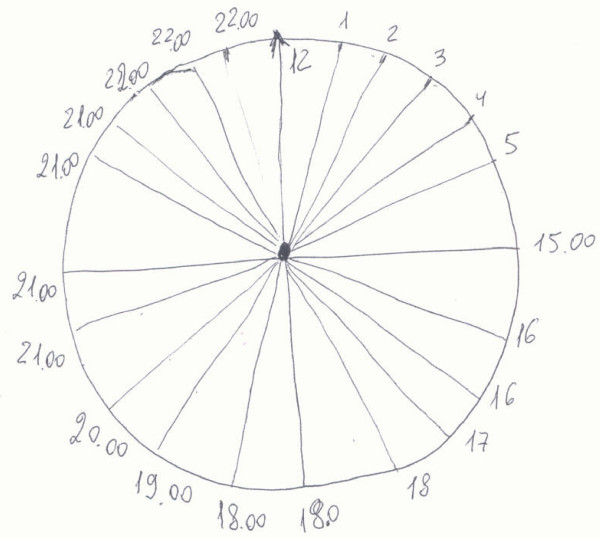
**Clock drawing test**.

During the first week of hospitalization, the major difficulty has been to establish a dialogue with her. She hardly communicated, she was very confused and she looked like she was experiencing visual hallucinations: her eyes were like following images in the space around her, and her hands were moving in front of her eyes, in a way similar as she had to get rid of webs. She couldn't speak Italian anymore, – although she was able to speak Italian before- but kept on understanding what we told her; she was still able to speak Russian, her mother language. Some symptoms could be reconnected to our diagnostic hypothesises, but, at the same time, others ruled out them [4-6]. During this period she took low doses, 3 mg/die, of Risperidone, an antipsychotic drug, without clinical improvement. During following days, the clinical development was very variable and characterized by fluctuations of the consciousness state, stereotypic movements, urinary incontinence and vomiting episodes. A significant episode occurred when we asked the patient to make her signature and she did it with syllable reduplications. This one and subsequent neuro-cognitive tests, such as the "clock drawing test" (fig. 2), suggested a neurological diagnostic hypothesis.

We requested a new neurological consultation, where the patient showed any peripheral neurological signs, but several cognitive symptoms as apraxia, disturbance in executive functioning and partial amnesia, beside aphasia.

A second Electroencephalography examination showed a slow basic activity in both brain hemispheres and marked focal epileptic type anomalies in right frontal area, with a tendency to spread on the left homologous region.

Focusing on inflammatory or infective disease, we sent the patient, forty days after the first – negative – TC, to a new neuroradiological evaluation, and the specialist chose to undertake Brain Nuclear Magnetic Resonance: it evidenced a large lesion in the right frontal lobe, a smaller lesion in the left frontal lobe, a large irregular halo of altered signal on both sides, probably attributable to perilesional oedema.

Most likely, a contrast CT would have highlighted such big lesions in a similar way, but MRI was first undertaken, thinking it was an inflammatory or infective disease.

After Brain Magnetic Resonance, the patient was quickly transferred to the neurosurgery unit.

### Neurosurgery Unit

**Figure 3:**
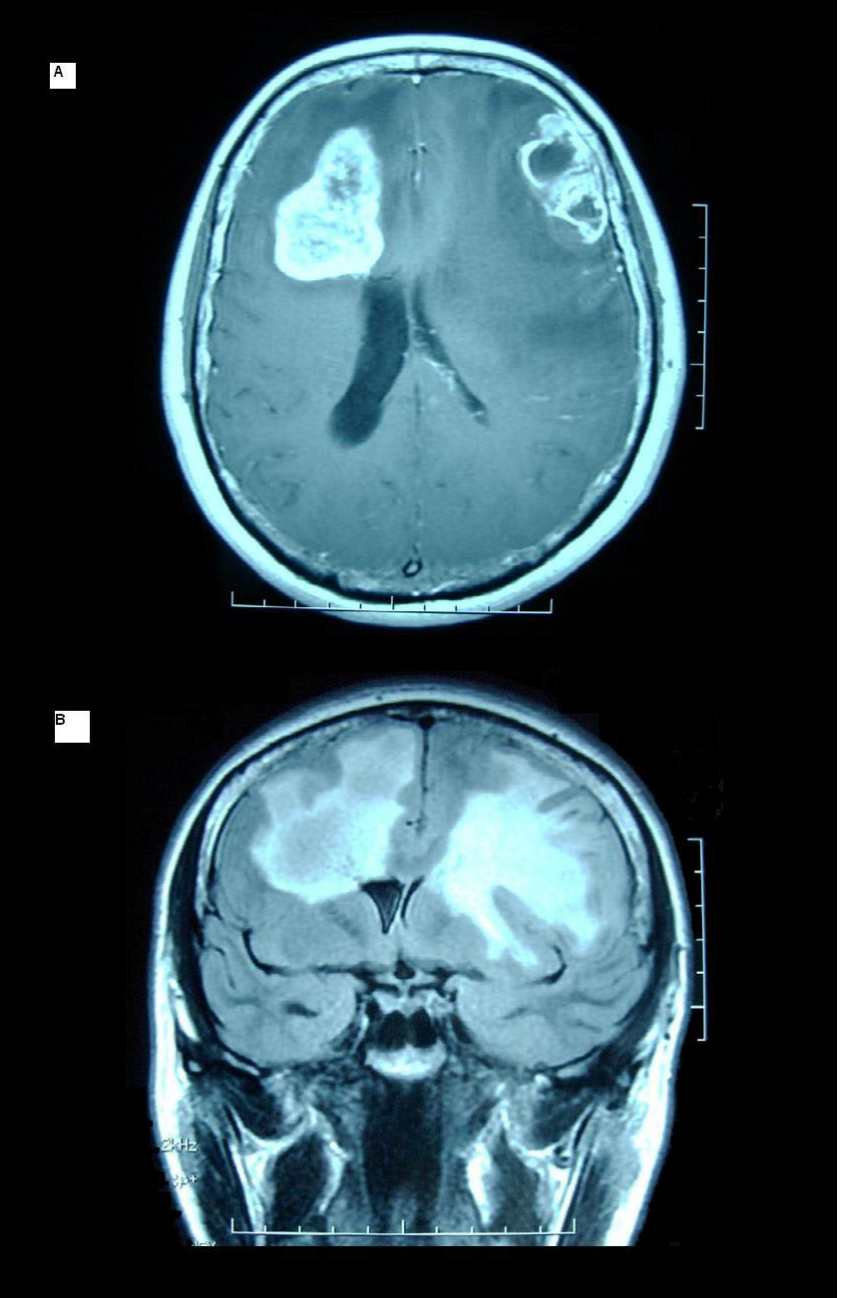
**A – Preoperative T1gado-axial Magnetic Resonance Imaging; B – Preoperative FLAIR-coronal Magnetic Resonance Imaging**.

A magnetic resonance imaging (MRI) showed two frontal focal lesions (4 × 3 cm on the left side and 5 × 4 cm on the right side) with peripheral contrast enhancement, surrounded by T1-ipointense and T2-hyperintense signal limited to frontal lobes (fig. Fig. 3). Functional sequences detected increased microvascular permeability and cerebral blood volume, suggesting neoangiogenesis.

Her neurological examination was characterized by lethargy, apathy, mutacism, visual hallucinations, impairment of 6^th ^right c.n. and papilloedema.

We administered antiedemigenic therapy with high doses of mannitol and dexamethasone.

**Figure 4:**
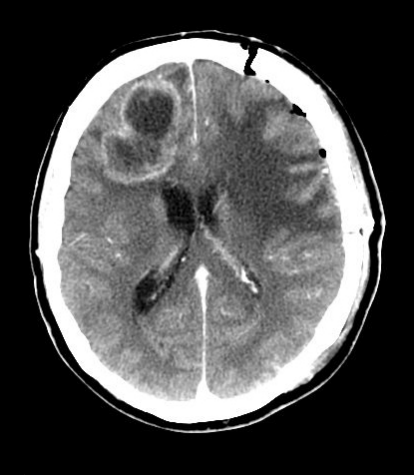
**Brain Computed Tomography performed 24 h after first surgical intervention**.

In consideration of the neuroradiological findings, of inability to distinguish between primary and metastatic lesions and of the absence of other primary lesions on the thoracic-abdominal computed tomography (CT) scans, we performed surgical removal of the left frontal lesion. Our choice to approach the left lesion was related to its important mass effect and cortical localization. The removal was radical as confirmed by the post-operative CT scans(fig. Fig. 4)

**Figure 5:**
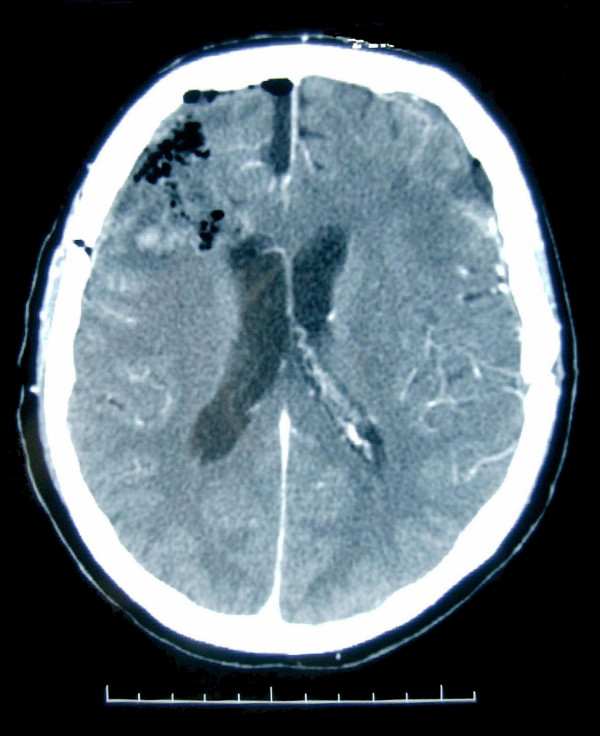
**Brain Computed Tomography performed 24 h after second surgical intervention**.

After surgery, we assisted to rapid awakening and a gradual improvement of apathy and mutacism. Glioblastoma was histologically defined and then we performed the total removal of the contralateral lesion. Also in this case, histology reported the presence of a glioblastoma. A post-operative CT confirmed the radical tumors excision (fig. Fig. 5).

Three weeks later the patient received limited field irradiation radiotherapy (40 Gray in 15 fractions over a period of 3 weeks) and temozolomide.

Two months later, the patient was able to perform her daily life activities with a mild disphoria (KPS – Karnovsky Performance Scale 100).

## Conclusion

Multicentric glioblastoma is a rare entity. The clinical presentation may develop extremely fast and can be characterized quite exclusively by cognitive symptoms; such symptoms can be very difficult to place, especially when focal neurological signs are not present. In this particular case, we couldn't expect a so relevant evolution in such short time.

"Psychiatric" patients are, often erroneously, considered difficult subjects because they are considered to need additional care: this may lead to underestimate symptoms and consequently a delay in diagnostic process.

In case of atypical psychiatric presentation, it should be taken into consideration neoplastic, inflammatory or infective causes.

Despite the absence of focal neurological signs and basal CT scan alterations, complementary imaging examinations, such as MRI and contrast enhancement CT, are necessary, especially when atypical symptoms are present and the conditions become quickly worse

## Consent

"Written informed consent was obtained from the patient for publication of this case report and accompanying images. A copy of the written consent is available for review by the Editor-in-Chief of this journal."

## Competing interests

The authors declare that they have no competing interests.

## Authors' contributions

MCTurola, MGNanni and GFF observed the patient during her psychiatric admission, discussed the case and the diagnostic program; ADV took care of the patient in her admission in neurological unit; RS and VR performed surgical procedures and followed the patient in the post-operative period. Each specialist wrote specific part of the clinical history, all together discussed the case and wrote abstract and conclusion. All Authors read and approved the final manuscript.
